# Life’s Energy and Information: Contrasting Evolution of Volume- versus Surface-Specific Rates of Energy Consumption

**DOI:** 10.3390/e22091025

**Published:** 2020-09-13

**Authors:** Anastassia M. Makarieva, Andrei V. Nefiodov, Bai-Lian Li

**Affiliations:** 1 Theoretical Physics Division, Petersburg Nuclear Physics Institute, Gatchina 188300, Russia; 2 USDA-China MOST Joint Research Center for AgroEcology and Sustainability, University of California, Riverside, CA 92521-0124, USA

**Keywords:** biota, energy, information, life, decay, environment, metabolic rate, ecosystem, civilization, forests

## Abstract

As humanity struggles to find a path to resilience amidst global change vagaries, understanding organizing principles of living systems as the pillar for human existence is rapidly growing in importance. However, finding quantitative definitions for order, complexity, information and functionality of living systems remains a challenge. Here, we review and develop insights into this problem from the concept of the biotic regulation of the environment developed by Victor Gorshkov (1935–2019). Life’s extraordinary persistence—despite being a strongly non-equilibrium process—requires a quantum-classical duality: the program of life is written in molecules and thus can be copied without information loss, while life’s interaction with its non-equilibrium environment is performed by macroscopic classical objects (living individuals) that age. Life’s key energetic parameter, the volume-specific rate of energy consumption, is maintained within universal limits by most life forms. Contrary to previous suggestions, it cannot serve as a proxy for “evolutionary progress”. In contrast, ecosystem-level surface-specific energy consumption declines with growing animal body size in stable ecosystems. High consumption by big animals is associated with instability. We suggest that the evolutionary increase in body size may represent a spontaneous loss of information about environmental regulation, a manifestation of life’s algorithm ageing as a whole.

## 1. Introduction

Modern civilization aims to provide an increasing number of people with decent living conditions. Keeping the civilization running requires continuous work on its maintenance. With the increasing reliance of the civilization on the information processing and computer power, the phenomenon of life began to be studied from the viewpoint of its information content [1,2,3,4,5,6]. Gorshkov [7] estimated the information processing capacity of the global biota from the rate of photosynthesis at $10^{35}$ bit s${}^{-1}$ assuming that the biota computes at maximum possible efficiency of the order of $kT\approx 10^{-21}$ Joules per bit [8,9,10,11,12]. This exceeds the global computing capacity of the modern civilization by about fifteen orders of magntude. Recent estimates of computing efficiency in protein synthesis supported the proposition that living systems are fairly close to the Landauer limit and many orders of magnitude more efficient than modern computers [13]. Per unit surface area, the biota consumes two orders of magnitude less power but performs ten millions more operations per second than modern supercomputers demonstrating a billion times greater computing efficiency [14].

An essential problem is the meaning of biological information [6,15]. Estimates of computing efficiency depend on the definition of calculation, i.e., what constitutes processing of one bit of information in biological systems. Gorshkov [7] considered that during photosynthesis all transitions of biological molecules from an excited to a ground state represent non-random genetically programmed processes; any such transition can be counted as processing of one bit of information. On the other hand, it is obvious that, for example in protein synthesis, the process of translation will proceed independently of whether the synthesized proteins are beneficial for the organism or disrupt its functioning (e.g., if these are proteins encoded by the coronavirus genome). Likewise, the cost of printing does not depend on the content of the book being printed.

The evolutionary theory focusing on the “survival of the fittest” does not provide clues to defining meaningful biological information, as the fittest is defined a posteriori as the best survivor. Information theorists, on the other hand, defined “the fittest” algorithm as the one making the fewest errors in calculations [2,3,16,17]. However, as long as the biological meaning of “calculation”remains undefined, the relevance of such algorithms and patterns associated with their artificial selection to real life is not straightforward.

Searching for more objective quantitative criteria of complexity, Chaisson [18], see also Chaisson [15,19,20], proposed to consider energy rate density (the rate of energy consumption per unit mass of the object). The ultimate rationale behind this proposition is apparently related to the fact that, when the rate of external energy consumption is zero, the systems come to the state of thermodynamic equilibrium (chaos). Thus, complexity and order should, at least under some conditions, relate to external energy flow. A similar approach was taken by Gorshkov and Makarieva [21], see also Gorshkov et al. [22], who pointed out that the density of memory cells grows with external energy flow in physical systems like atmospheric eddies.

However, while Chaisson [18] considered some evidence for life systems that presumably supported the idea of a greater evolutionary complexity associated with a higher energy consumption per unit mass, Gorshkov and Makarieva [21] emphasized that the inanimate and animate worlds principally differ, with universal energy consumption rates common for most life forms. The key difference between living and non-living systems lies in the fact that living systems do not arise spontaneously anew in whatever external energy fluxes, but only arise as copies of other living systems. The complexity of living systems appears unrelated to the degree of orderliness of external energy that supports them. It is maintained by the information written in life itself; this information encodes how natural selection of living objects operates and the criteria of “the fittest”.

The biotic regulation concept developed by Gorshkov [23] describes how orderliness of living systems is impossible to maintain without the program of life containing the information about how to stabilize the environment in a suitable for life state—where the program of natural selection will ensure the long-term persistence of life’s algorithm. For the program of biotic regulation to remain competitive, the biotically regulated environment should be highly non-equilibrium and physically unstable without the biotic control. In a different context, but on a converging line of thought, Langton [3] suggested that *“evolution reflects the process by which life has gained local control over a successively greater number of environmental parameters affecting its ability to maintain itself at a critical balance point between order and chaos”.*

Since the concept of the biotic regulation of the environment is less known in the English-language literature [24], here we give a brief outline of its major ideas and how they relate to the on-going debates in the fields of evolution, ecology, and information theory (Section 2). In Section 3, we re-analyze and revise the estimates by Chaisson [15,18,19,20] to show that there is no support to the statement that more advanced evolutionary forms of life possess a higher rate of mass-specific energy consumption. In Section 4, we consider the energy consumption rate per unit surface area and discuss how the distinct size-related patterns on individual and ecosystem levels provide insights into life’s stability and persistence. The larger animals with their low surface-to-volume ratio have a low regulatory potential, low capacity for processing of environmental information, and may disrupt ecosystem functioning. In Section 5, we discuss how the high energy consumption by modern civilization is unlikely a sign of high orderliness but a sign of unsteadiness warning of a potential collapse. In the final Section 7, we discuss how life’s major organizing principle, persistence via ensuring genetic and environmental stability, gives the meaning to biological information. We discuss how big disruptive animals, humans, could nevertheless culturally evolve into the guardians of the biosphere and prolong its existence using the scientific analysis of the laws of life functioning and adopting behaviors respecting those fundamental laws.

## 2. Biotic Regulation of the Environment

### 2.1. The Genome, Organism, and Environment Triade

**Figure 1:**
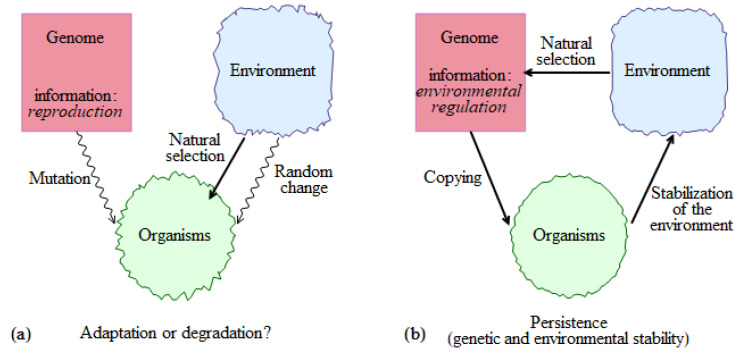
Conceptualization of life in the more conventional “adaptive” interpretation (**a**) and within the biotic regulation concept (**b**). (**a**) Main genetic information is about how to reproduce. Mutations that accumulate during reproduction (copying of genetic information) serve as an ultimate source for adaptive genetic diversity. The environment undergoes random changes. In a given environment, some individuals survive better than others; this represents natural selection. (**b**) Main genetic information is about how to stabilize the environment in an optimal state. The genetic information written in quantum molecular memory cells can be copied without errors (ageing). Classical objects (organisms) encoded by this information stabilize the environment compensating for external and internal perturbations. The optimal environment is the one where the genetic information that encodes this environment features maximum competitiveness. In other words, life encodes and maintains an environment where the algorithm that does so possesses maximum fitness.

A more complete description of the biotic regulation concept can be found in Gorshkov [23] and Gorshkov et al. [25], see also Gorshkov and Makarieva [26,27,28]. Here, we introduce its main propositions at the simplified background of the more conventional ideas about life, evolution, and environment (Figure 1a). The key genetic information of life is assumed to be that about reproduction. Copying of genetic information occurs with errors (mutations), which ensure that all organisms in a population differ (genetic diversity). In any given environment, some organisms reproduce better than others, their genetic information predominantly propagates. The environment may experience random changes, and the criteria of selection it imposes on the organisms, change as well. This causes the genetic information of a species change with time, which on a larger scale constitutes biological evolution.

This qualitative picture does not explain the long-term persistence of life and leaves plenty of room for alternative visions. Indeed, as mutations accumulate in the course of copying of genetic information, some individuals with erroneous copies will, in a given environment, reproduce better than others, so there will always be a natural selection of some sorts. However, selection based on *relative* fitness will not guarantee that the *absolute* fitness, i.e., the ability of organisms to produce a sufficient amount of healthy progeny, will not decline with time. Indeed, while it has become clear that, in the human population, natural selection is too weak to prevent accumulation of mutational load [29], it is not clear what general principles would guarantee the necessary efficiency of selection for other species of the biosphere. Besides genetic degradation, another opportunity for life to go extinct comes from the unpredictable, uncontrollable, and unfavorable environmental changes.

A logical way to account for life’s persistence without appealing to a lucky chance is to assume that life’s basic information kit includes, as its key component, the information about how to regulate the external environment (Figure 1b). Governed by this program, living organisms impose a stabilizing impact on the environment keeping it in a state optimal for life’s functioning. The notion of optimality presumes that, in this environment, those individuals that perform environmental regulation must be “the fittest”. This condition of a simultaneous genetic and environmental homeostasis imposes strict constraints on the properties of the optimal environment.

A comprehensive quantitative analysis of these constraints represents a distinct feature of the biotic regulation concept in comparison to other propositions of life’s stabilizing environmental impacts, including the Gaia hypothesis [30]. Essentially, the optimal environment must be strongly non-equilibrium, such that, if left without biotic control, it would rapidly degrade to a state decreasing fitness of all organisms. This condition ensures that a mutant with eroded program of environmental regulation, even if initially competitive in the optimal environment, will be unable to spread globally as it will have rapidly destroyed its life-important resources.

Evolution and environmental change are considered as first-order corrections to the main picture of life’s homeostasis (Figure 1b), see also Haldane [31]. Evolution occurs in the direction of a more efficient program of environmental regulation. Paraphrasing a well-known saying about evolution, one can say that nothing in life sciences makes sense except in the light of life’s persistence.

### 2.2. Quantum and Classical Nature of Life

How can living organisms ensure persistence of non-equilibrium environmental conditions? The information about characteristics of the optimal environmental state has to be stored and copied. Since the environment of life is macroscopic, it represents a classical object that ages—changes irreversibly with time. Copying of classical objects is not possible without information loss; they undergo decay and are different at any point of time. Thus, the information about environmental conditions, as well as all other life’s information, must be written using quantum carriers—objects that do not age and possess the property of identity.

In most biological species, the information about life’s complexity is written in the polymer molecules of deoxyribonucleic acid (DNA). Total information written in these molecules constitutes a species’ genome. Once this information is lost in extinct species, it cannot be recovered. From paleodata, we know about the dinosaurs, mammoths, and other extinct species. We also know about species driven to extinction by humans—Steller’s cow, the dodo bird, the passenger pigeon, the elephant birds, and others. Their genomes are irreversibly lost and irrecoverable. No extinct species ever came into existence again. The paleodata also indicate that the majority of extant species did not change morphologically for millions of years [32]. It logically follows that their genetic program remained invariant as well [33]. Furthermore, all extant life shares a common ancestor, i.e., during the four billion years of life existence there was only one origin of persistent life.

The genetic information can only be copied, not created anew. Copying requires energy expenditures that come from the external environment. Information of the polymer DNA molecules is written with use of four molecular letters—nucleotides. These four letters can bind into complementary pairs to form the double-stranded four-letter DNA molecule. As this molecule is to be copied, the double DNA strand unravels, and, on each of the two strands, the complementary part is synthesized. In the result, two identical DNA molecules are formed of two different cells belonging to one and the same or different individuals of the same species.

During the copying process, errors may occur partially erasing the information DNA carries. These copying errors obey the well-known pattern of radioactive decay. In a characteristic time period (the half-life time), one half of the initial number of the copied molecular objects acquire some errors. The other half remains error-free and *identical to the previous generation*. This identity is what constitutes the quantum nature of life. If life did not possess this property, i.e., there were no identical copies produced during copying, it would have been impossible to sustain the orderliness of life.

With the probability of point mutation affecting a given nucleotide pair of about $\nu =10^{-10}$ per cell division [34] and division frequency of the order of $\tau^{-1}=q/K$, where q=1 W kg${}^{-1}$ is the universal mass-specific metabolic rate and $K=4\times 10^{6}$ J kg${}^{-1}$ is the energy content of living matter [28], the genetic information of life would have melted completely in about τ/ν≈ one billion years. The observed level of tolerated genetic polymorphism in biological species, about one mutation per $10^{3}$ nucleotides, indicates that a loss of function occurs when about one thousandth of the total amount of information has been lost. This gives a time scale of genetic decay of about one million years. Incidentally, this is close to the mean time of species’ existence [35,36]. If the program of life contains information about how to recognize and delete the erroneous copies from the population, then it could be possible to maintain life’s orderliness for an unlimited period of time—or at least to prolong its existence far beyond the half-life time of genetic information. The necessary condition is that erroneous copies should be deleted faster than they reproduce themselves. Again, this condition constrains the properties of the optimal environment where life could persist.

Recognizing and deleting the erroneous copies cannot be realized on the quantum level of DNA molecules. Classical macroscopic organisms are required that are able to consume energy and matter from the environment and function on the basis of the genetic DNA program. Namely, in a population of such macroscopic classical individuals that all age, the program of recognizing and deleting the erroneous information copies can be realized. It is the most complex part of the genetic program itself. This statement is key to the biotic regulation concept. Competitive interaction and natural selection are not merely imposed on the population by an external environment being passive outcomes of the organism–environment interaction (cf. Figure 1a). Instead, competitive interaction and natural selection represent the key part of the genetic program itself.

This property is not captured in the studies of complexity maintenance by cellular automata e.g., [2,3,17]—there the program of selection is external and unrelated to the properties of the algorithm it selects. For example, in the well-known experiment of Mitchell et al. [17], selection was set to favor the algorithms that correctly performed a certain calculation. This selection strategy got no feedback from the selected algorithms. More generally, among the many aspects studied by information theory in biological systems [37], the crucial aspect of how life interacts with its environment is practically missing.

### 2.3. Energy Flow Slow-Down and Life’s Persistence

Macroscopic physical processes in the Earth’s environment do not copy each other but arise anew triggered by external energy flows, of which on Earth the most important one is the solar radiation. All life and civilization are maintained by the ordered solar radiation which is close to the blackbody radiation with temperature $T_{S}=6000$ K. The Earth receives solar energy in the form of massless photons. After these photons have perturbed the ground states of molecules on Earth, and the excited states relaxed back to the ground state, the solar photons turn to thermal photons with mean temperature $T_{E}=300$ K. The power of solar radiation absorbed by the Earth’s surface undergoes decay to thermal radiation via a multitude of diverse channels provided by the living systems, civilization, and the inanimate nature of winds, oceanic currents, storms, hurricanes, and tornadoes.

As the black body radiation consists of photons with mean energy kT, where *k* is the Boltzmann constant, each solar photon with mean energy $kT_{S}$ decays into approximately $T_{S}/T_{E}=20$ thermal photons of the Earth with mean energy $kT_{E}$. This decay of solar photons into thermal photons shape all events and determine the time of all processes on the Earth’s surface. In most fields of theoretical physics, it is assumed that time is isotropic, which presumes that its direction can be reversed. The equations of motion in theoretical physics do not contain information about the decay of excited states of matter. These equations describe transitions of kinetic to potential energy and back for the two known long-range interactions, electromagnetic and gravitational. In the gravitational interaction, time defines the periods of potential-to-kinetic energy transitions, as in ideal pendulum, or rotation of interacting masses over elliptic orbits that characterize the energy of their interaction. In neither of these cases does anything change in the considered processes except the number of oscillations or rotations grows counted from an arbitrary initial state. This number is referred to as time. Thus, defined time does not have a direction and it can be reversed, i.e., it conforms to the condition of isotropy. The considered processes change if only one takes into account dissipation and decay of excited states.

A major achievement of quantum physics consists in the discovery of the identity of the ground and excited states of atoms, molecules, and atomic nuclei. Two quantum objects being in the same states cannot be distinguished from one another. All excited states conform to the law of half-life time decay: starting from a certain initial number of identical excited states, since the so-called half-life time counted using the number of non-dissipating oscillations or rotations, the number of the excited states diminishes by half. The remaining excited states are identical to the initial excited states. The second half undergoes spontaneous or induced decay. Induced decay diminishes the half-life time, but does not change the pattern: there always remain states that have not undergone decay and are identical to the initial states. Those states that have not undergone decay do not change (“wear”) or age. Only those states that have undergone decay age and wear. The half-life time is a fundamental constant equal for all specific states irrespective of their “age”. Life is capable of selecting quantum programs with the smallest or optimal half-life time in the environment.

Let us define time as the number of arbitrarily chosen non-dissipating oscillations or rotations that have occurred from the moment when a certain initial amount of ordered states capable of undergoing decay has been prepared, to the moment when they have all undergone decay. If a new excited state has not been prepared by then, the time disappears. The direction of time is uniquely related to the diminishing number of the excited states, and thus automatically relates the cause and consequence. The decay of an excited state is accompanied by release of energy that can be routed along the additional decay channels. The power of a process is equal to the energy of the half of the initial states that have undergone decay divided by the half-life time. An event is the process of decay of an ordered state capable of undergoing decay. If there is no decay, there is no time and no events. If the Sun had been sending the same radiation power but in the thermal part of the spectrum corresponding to $T_{S}=T_{E}=300$ K, the Earth would have remained as warm as it is now, but the decay of solar radiation would have been impossible. No processes related to this decay, including the process of life, would be possible. Time would have stopped.

The ordered states that can undergo decay are slowly prepared at the expense of the energy of solar radiation. In the absence of a program governing how they will decay, their decay occurs explosively and the diversity of the possible channels for energy transformation is limited. Rare classical macroscopic physical processes, like wind, rain, storms, hurricanes, and tornadoes, “die” and arise anew. They consume the available energy in an explosion-like manner and discontinue until new stores of energy arise in the environment. Molecular quantum copying sometimes spontaneously appears in the inanimate nature at the expense of previously accumulated energy which is likewise spent in an explosive manner (for example, in chemical reactions with catalysts or nuclear chain reactions). Such reactions do not lead to the formation of classical objects that recognize and delete erroneous copies. The explosion-like ordered processes in the inanimate nature discontinue as soon as the energy stores have been depleted, after which all objects that are being copied disappear.

The combination of classical and quantum processes to ensure persistence uniquely differentiates life from the inanimate world. To account for the apparent absence of life elsewhere in the Universe, Chopra and Lineweaver [38] proposed that life may originate relatively often but evolves too slowly to gain control over the planetary environmental conditions. The life-compatible conditions are otherwise unstable and quickly deteriorate driving the “non-regulating life” to extinction. In comparison, from the biotic regulation viewpoint, a “life” that is not capable of controlling conditions perpetuating its persistence, is not a life. As we discussed, self-copying (reproduction) and metabolism (energy consumption) are common to many processes in the inanimate world.

Life’s persistence corresponds to a genetic program that suppresses the explosive energy consumption. A good example is provided by how the biota handles the phase transitions of water on Earth. Water vapor evaporation under the influence of solar radiation is a slow distributed process. Condensation of water vapor, in contrast, is governed by the vertical velocity of the ascending air (the faster it ascends, the faster it cools and moisture condenses), so it may occur at an arbitrarily high rate that exceeds by orders of magnitude the rate of evaporation. Such explosive-like release of energy takes the form of hurricanes and tornadoes that quickly deplete all water vapor previously accumulated in the atmosphere [39], Table 1. Over natural undisturbed forests on land, where the hydrological cycle is under biotic control, the spatial and temporal fluctuations of precipitation and wind are strongly suppressed [40,41,42]. The biota of the forest plants, fungi, and bacteria on land is able to slow down the rate of water vapor condensation sucking the ocean-evaporated moisture to land. The rates of evaporation and condensation become more equal, and there appears an opportunity of an uninterrupted time that follows the decay channels generated by life.

Civilization slowed down the decay of uranium in nuclear fission reactions and uses them for energy production (Table 1). Accidental loss of control over the slow-down leads to explosions at the nuclear power plants (Chernobyl, Fukushima). Civilization did not succeed in slowing down the decay of the prepared initial state in the thermonuclear fusion reaction. Consumption of fossil fuels by civilization has been exponentially increasing demonstrating explosion-like dynamics [43]. Destruction of the forest cover by civilization likewise proceeds in an explosion-like manner, with losses of the order of 100% over one tree life span [44].

The biomass of the immobile life persists due to the equality between the rates of synthesis and decomposition of organic matter by plants, bacteria, and fungi on land and in the ocean. Large animals including humans are able to destroy the biomass of plants at high rates that significantly exceed the slow rate of photosynthesis. The uninterruptedness of the time of life has been maintained because the share of biomass consumption by big animals remained small. If all synthesized biomass is consumed, in an explosion-like manner, by large animals and man, the uninterruptedness of time can be broken and the stability of life on Earth destroyed. Therefore, the uninterrupted time on Earth is determined by life in the decay channels that life itself regulates, i.e., it is a consequence of the existence of life. (Only within the volume of the Sun the thermonuclear reaction is slowed down at the expense of radiation emission that prevents the star from the gravitational collapse. It is the only slow-down of energy flow that is independent of life on Earth.) In what is to follow, we consider these statements in greater detail.

## 3. Volume-Specific Energy Rate Density

In a search for quantitative criteria of life’s orderliness and complexity, a proposition was put forward by Chaisson [18], see also Chaisson [15,19,20] that evolutionary progress and an increase of complexity with time in both animate and inanimate nature is reflected in the magntiude of the energy rate density. Assuming, additionally, that *“complexity generally increases with evolution”* [19], higher energy rate densities in organisms that appeared later in the evolution were interpreted as a correlation between energy rate density and complexity.

Here, we reconsider these estimates and their more recent interpretations [13] to show that there is no evidence for any systematic large-scale changes in mass- or volume-specific energy rate density in the course of biological evolution. In the biological context, individual rate of energy consumption is called metabolic rate, so these terms will be used here interchangeably. In addition, since the density of living matter is close to the density of liquid water $\rho =10^{3}$ kg m${}^{-3}$, mass *m* and volume *V* in living objects are related as m=ρV, thus mass- and volume-specific rates differ by a constant ρ.

**Figure 2:**
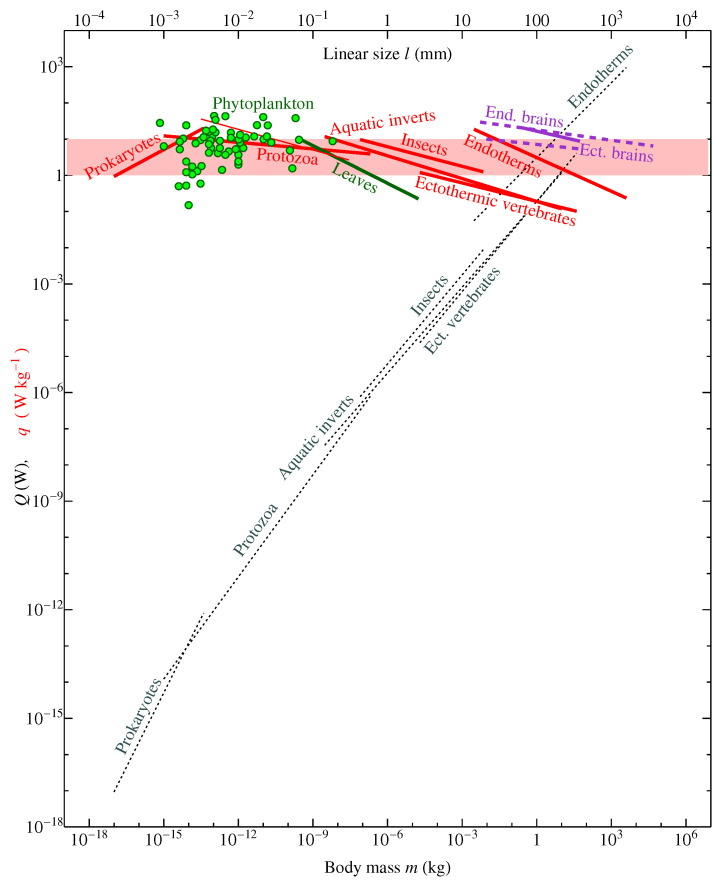
Metabolic portrait of life in terms of whole-body metabolic rate *Q* (W) and mass-specific metabolic rate *q* (W kg${}^{-1}$) of individual organisms and their brains. Pink shading denotes the proposed optimal range for life’s functioning, approximately between 1 and 10 W kg${}^{-1}$ [58,62]. Red solid lines are the allometric dependencies of *q* on body mass *m* for groups of heterotrophic species from Table 1 of Makarieva et al. [58], gray dotted lines are the same dependencies for Q=qm. The thinner red line for Protozoa represents starved cells from Ikeda [63] Table S1-1. Green circles are values for phytoplankton species (cyanobacteria and microalgae) from the Supplementary Information of Makarieva et al. [58]. Green solid line is the mass-specific rate of dark respiration in green leaves depending on leaf thickness *l* calculated from the data of Wright et al. [64]. Linear size *l* (upper horizontal axis) and body mass *m* (lower horizontal axis) relate as $l={\left(m/\rho \right)}^{1/3}$. The dependencies for the mass-specific brain metabolic rate in ectotherms and endotherms on body mass (measured in vivo, violet solid line, and estimated indirectly, violet dashed lines) are constructed using the data of Mink et al. [65]. See Section 6 for details.

Figure 2 Chaisson [15] summarized his previous estimates, according to which the more complex plants with their metabolic rate of about 1 W kg${}^{-1}$ evolved from the more primitive protists that presumably had a lower metabolic rate of about 0.1 W kg${}^{-1}$. The latter figure was obtained by Chaisson [18] (p. 138), see also Chaisson [20], for the biosphere as a whole by dividing the global rate of photosynthesis *F* by global plant mass. Using modern data, this estimate can be obtained as

(1) $$q_{b}=\frac{F}{M}K_{C}\times \frac{1\;\mathrm{kgC}}{10\;\mathrm{kg}}=6\times 10^{-2}\;W\;{\mathrm{kg}}^{-1}.$$

Here, M=450 GtC is the total plant mass in the biosphere in carbon units [46], F=200 GtC yr${}^{-1}$ is the global gross primary productivity, $K_{C}=42\times 10^{6}$ J (kgC)${}^{-1}$ is the energy content of organic matter [58]. Chaisson [20] used a lower value for plant biomass, 1200 Gt instead of 450 GtC ≈ 4500 Gt and a lower value for the biosphere energy consumption (10${}^{21}$ erg s${}^{-1}$ = 10${}^{14}$ W instead of $FK_{C}=3\times 10^{14}$ W) to obtain $q_{b}=9\times 10^{-2}\;W\;{\mathrm{kg}}^{-1}$.

Chaisson [15] Figure 2, see also Table 3 of Chaisson [19], Figure 6 of Chaisson [20], attributed $q_{b}=0.1$ W kg${}^{-1}$ to protists and referred to it as *“valid for the great majority of Earth’s lower plant life”*. In Table 3 of Chaisson [19], it is stated that examples of such low energy rate density is provided by *“phytoplankton, algae.”*

This is incorrect. The low value of $q_{b}$ (1) is due to the fact that most part of plant biomass is wood, which is metabolically inactive e.g., [46,59]. Wood performs mechanical functions not related to the biochemical energy conversion. To estimate the energy consumption of plants per unit wood mass is similar to estimating the energy consumption of birds per unit mass of their nests or energy consumption of the civilization per unit mass of buildings. In the ocean, plants lack such metabolically inactive parts. Global biomass of oceanic phytoplankton $M_{p}\approx 1.3$ GtC is several hundred times lower than *M*. With gross primary production of the ocean about $F_{p}\approx F/2\approx 100$ GtC yr${}^{-1}$, mean mass-specific photosynthetic rate of phytoplankton is

(2) $$q_{p}=\frac{F_{p}}{M_{p}}K_{C}\times \frac{1\;\mathrm{kgC}}{10\;\mathrm{kg}}=10\;W\;{\mathrm{kg}}^{-1}.$$

This value for phytoplankton is significantly higher than the values quoted by Chaisson [15] [Figure 2 and references therein] for the higher plants like gymnosperms and angiosperms (0.5–1 W kg${}^{-1}$). It falls on the upper range of mass-specific metabolic rates of life as a whole (Figure 2) and coincides with the metabolic rate of human brain, which in young healthy adults constitutes around 1.3 μmol O${}_{2}$ min${}^{-1}$ g${}^{-1}$ [60,61], which corresponds to 10 W kg${}^{-1}$ assuming 20 J (ml O${}_{2}$)${}^{-1}$.

Direct measurements of metabolic rates in phytoplankton and protists confirm that these organisms do not have lower metabolic rates than the higher plants and animals (Figure 2). Notably, even if we calculate the metabolic rate of higher plants per unit mass of their photosynthetic tissue, still for plants on land we obtain a mean figure of about 1 W kg${}^{-1}$, since the biomass of green leaves is about ten times larger than phytoplankton biomass Supplementary Information Bar-On et al. [46], while gross primary productivity on land is about the same as it is in the ocean. This is also consistent with direct estimates of respiration rates in green leaves (Figure 2).

In a more recent analysis, Kempes et al. [13] recognized that there is no consistent change of mass-specific metabolic rate across all life. Kempes et al. [13] wrote that while *“multicellular life obeys a certain economy of scale: as organisms grow larger, the metabolic rate required to support a unit of mass is decreasing and larger mammals support more tissues for the same amount of energy”*, more recently *“it has been observed that this scaling relationship is not preserved across all the taxa of life.”* Indeed, Makarieva et al. [58] established that *“mean metabolic rates of major taxonomic groups...converge on...a remarkably small range compared with the 4000- to 65,000-fold difference between the mean metabolic rates of the smallest and largest organisms that would be observed if life as a whole conformed to universal quarter-power or third-power allometric scaling laws.”* The dataset of Makarieva et al. [58] included a large compilation of prokaryote metabolic rates that had not been previously analyzed. Namely, these data for the smallest organisms allowed the generalizations about the metabolic portrait of life to be made.

Kempes et al. [13] did not cite the study of Makarieva et al. [58], but referred to DeLong et al. [66] who used the data of Makarieva et al. [58] for bacteria, converted them from mass-specific to whole-body basis and observed that the scaling exponent in bacteria, listed in Table 1 of Makarieva et al. [58], becomes greater than unity when the allometry is expressed per whole-body mass. Converting those data back to the mass-specific basis, Kempes et al. [13] discussed how the scaling exponent changes with growing body size of the organisms, noting that *q* first grows with size in prokaryotes, then it remains relatively constant in protists and then declines in larger organisms (Figure 2).

However, Makarieva et al. [58] emphasized that the methodological inaccuracy of measuring the link between organismal size and metabolic rate is profoundly different in the unicellular versus the multicellular organisms. In unicells, especially in prokaryotes, it is common to measure metabolic rate of a unit mass (e.g., taking 1 g dry mass of the bacterial suspension). Cell size is not simultaneously measured. Makarieva et al. [58] see Supplementary Information retrieved cell sizes from independent sources, often estimating cell mass from linear cell size. This procedure does not impact the value of mass-specific metabolic rate, but it should profoundly impact the dependence between mass-specific metabolic rate and cell size, especially given that the size of bacterial cells can change depending on their nutrition status (growth, starvation, etc.). This source of error is absent in larger organisms, where metabolic rate is commonly measured on a whole-body basis simultaneously with the body mass.

DeLong et al. [66] and Kempes et al. [13] did not assess the uncertainty associated with the poorly known cell sizes of unicellular organisms; at the moment, their conclusion about the difference in the scaling exponents between prokaryotes, unicellular eukaryotes, and higher organisms lacks statistical significance and cannot be meaningfully interpreted. A more up-to-date analysis of Ikeda [63] controlling for the physiological state in Protozoa indicates a decline in mass-specific metabolic rate with growing body size that is not distinguishable from what is observed in the groups of larger organisms (Figure 2, thin red line).

In summary, given that the low $q_{b}$ (1) assumed to characterize the lower life forms in the analyses of Chaisson [15] and previous work appears to be a miscalculation, while phytoplankton $q_{p}$ (2) falls instead on the upper border of all life energy rate densities, little support remains for a progressive increase in energy rate densities or any other consistent change in metabolic allometry with evolution (Figure 2), see also analyses by Ikeda [63], Johnson et al. [67], Kiørboe and Hirst [68], Hatton et al. [69].

Instead, since within evolutionary close groups of organisms the mass-specific metabolic rate declines with growing body size, while evolution tends to produce larger body sizes from smaller ones, the more recently evolved bigger forms within a group face the danger of leaving the life’s metabolic optimum range of 1–10 W kg${}^{-1}$ [58]. Once this happens, the bigger forms with low *q* could lose competitiveness and be forced out from the biosphere if a new group in the same body size interval evolves that has managed to elevate its rate *back to the optimal range.* In this context, Chaisson [15]’s proposition that endotherms who elevated their *q* rates above those of the similarly sized ectotherms might have been more evolutionarily advanced, at least in some aspects, remains meaningful.

**Figure 3:**
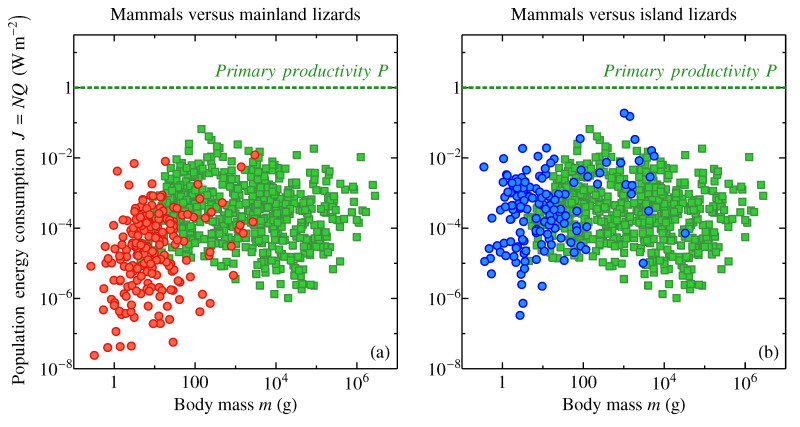
Energy consumption by populations of mammals (green squares) and mainland (**a**, red circles) and island (**b**, blue circles) lizards. Data for lizards and mammals are taken from Novosolov et al. [70] and Hatton et al. [69], respectively, see Methods for details. In addition, a characteristic value of net primary productivity (dotted line) P=1 W m${}^{-2}$ is shown.

Figure 3 displays the interplay between metabolic optimality and ecological dominance by comparing two groups of species, lizards [70] and mammals [69,71]. The green squares indicate population energy consumption of several hundred populations of mammalian species world over (mostly mainland) with a geometric mean of $3\times 10^{-4}$ W m${}^{-2}$ and half of all values confined between $0.9\times 10^{-4}$ and $12\times 10^{-4}$ W m${}^{-2}$. (Population energy consumption *J* is equal to population density (individuals per unit area or inverse area per one individual) multiplied by individual metabolic rate *Q*, J=NQ, see Methods). The red circles in Figure 3a show 198 lizard populations on the mainland, i.e., where the reptiles have to co-exist with mammals. The geometric mean energy consumption rate of a mainland lizard population is $2\times 10^{-5}$ W m${}^{-2}$ with half of all values confined between $0.4\times 10^{-5}$ and $14\times 10^{-5}$ W m${}^{-2}$. The population energy consumption by mammals is more than an order of magnitude higher than that of mainland lizards. On the ecosystem level, lizards are precluded from claiming the same amount of energy resources as mammals.

Things turn different on isands, where the competition with mammals is significantly reduced [70]. The blue circles in Figure 3b show population energy consumption in 148 island populations of lizards—compared to the mainland, it rises to $3\times 10^{-4}$ W m${}^{-2}$ with half of all values confined between $0.7\times 10^{-4}$ and $16\times 10^{-4}$ W m${}^{-2}$. Remarkably, the geometric mean values of population energy consumption in mammals and island lizards practically coincide. These data are consistent with the idea that the metabolically suboptimal lizards lose to mammals in the competition for ecosystem energy resources but re-gain their share of resources where this competition weakens or disappears altogether.

During evolution, the endotherms could have forced out some of the ectotherms with suboptimal metabolic rates (Figure 2) from their ecological niches causing their extinction—either by themselves or at the backstage of major environmental cataclysms. (For example, the metabolically ’sub-optimal’ dinosaurs, whose metabolic levels continue to be debated, see e.g., [72,73,74] and references therein, could have been outcompeted by the evolutionarily young metabolically optimal mammals.) Figure 3 illustrates a “metabolic footprint” of how this could have happened. Further insights into ecological dominance and metabolic optimality are provided by consideration of individual surface-specific energy consumption rates as dependent on the organism’s body size.

## 4. Surface-Specific Energy Rate Density at Individual versus Ecosystem Level

**Figure 4:**
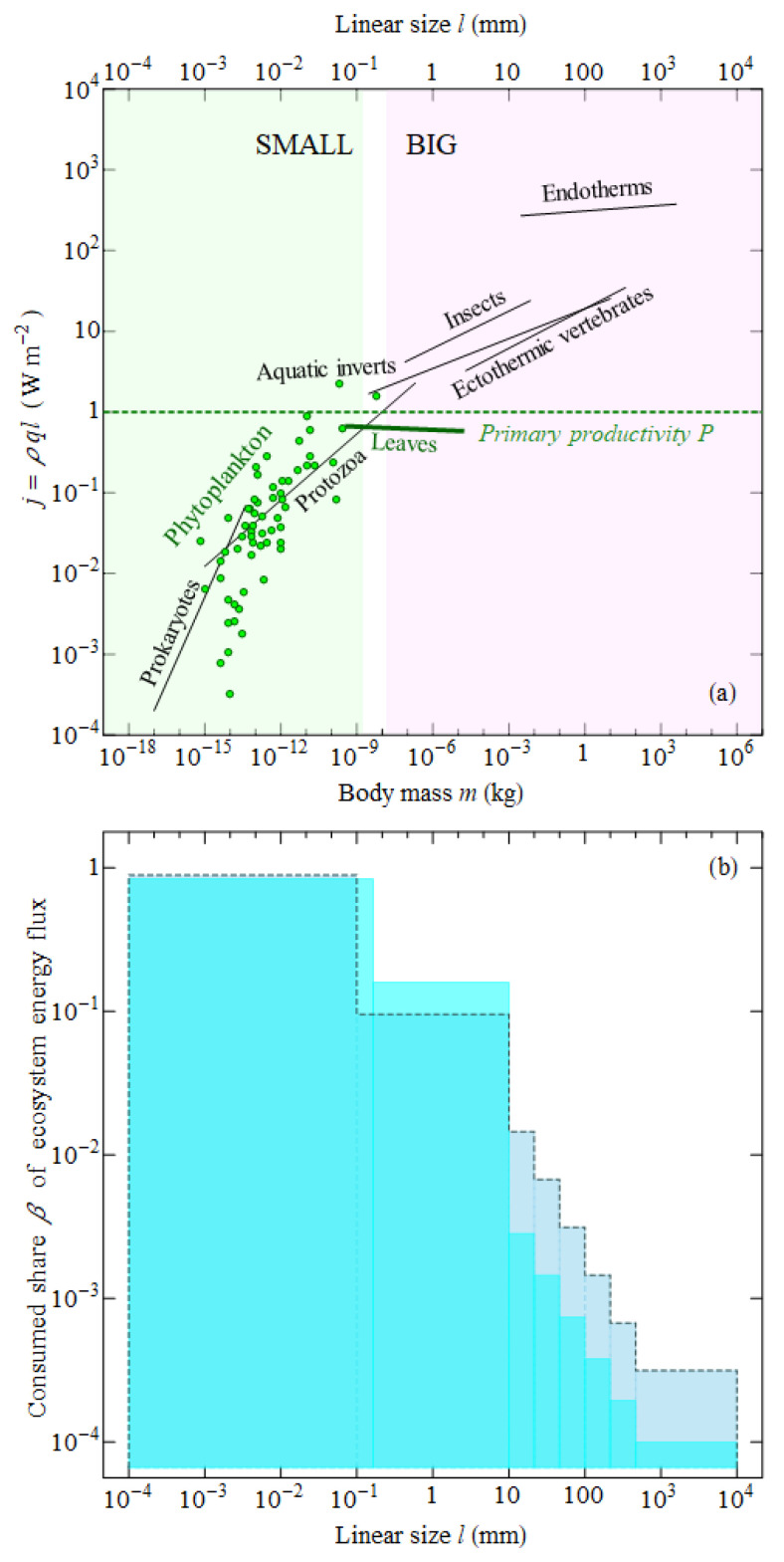
Surface-specific energy rate density on individual and ecosystem level. (**a**) individual surface-specific metabolic rate j=ρql (energy consumption per unit area of the body projection on the ground surface) for the organisms shown in Figure 2. The dashed line shows a characteristic value of primary productivity P=1 W m${}^{-2}$; (**b**) share β of ecosystem energy flux consumed by heterotrophs from a given body size interval in stable ecosystems on land (dashed contour histogram) [28,62] and in the ocean (solid contour histogram) [75]. The lower β values for the largest organisms in the ocean compared to land might be related to over-fishing, which reduced fish biomass in some body size intervals by at least an order of magnitude see, e.g., [76]. Note the logarithmic scale of the vertical axis in (**b**).

If, generally, the volume- and mass-specific metabolic rate *q* is size-invariant across life (Figure 2), then the surface-specific metabolic rate (or surface-specific energy rate density) $j=Q/S=q\rho l^{3}/l^{2}=\rho ql$, where *S* is the area of the organism’s projection on the ground surface, should grow linearly with linear size *l* of the organism. The flux of energy via the body surface of the smallest organisms like 1 μm bacteria is about $10^{-3}$ W m${}^{-2}$, while the biggest organisms like endotherms consume up to $10^{3}$ W m${}^{-2}$ (Figure 4a). In between lie the characteristic values of net primary productivity *P*∼1 W m${}^{-2}$ that plants generate using the energy of solar photons. The critical linear size $l_{c}=P/\left(\rho q\right)$∼$10^{-3}$ m divides all life into the small and the big, the immotile and the locomotive, the ecologically sustainable and the ecologically unstable [28,62].

Organisms of linear size smaller than $l_{c}$ consume less power than the biosphere is able to generate, j=ρql<P, per unit area of their projection on the Earth’s surface (∼$l^{2}$). Such organisms do not need to move actively or destroy live plants. They can satisfy their energetic needs by sitting and waiting for the dead plant parts falling down to become their food. Like plants themselves, such organisms can form a continuous cover, e.g., in the form of bacterial mats or fungi mycelia.

Conversely, species larger than the critical body size cannot sit and wait. They require more food per unit area per unit time than the biosphere is able to produce. Such organisms have to move collecting biomass synthesized by plants over a large home territory *H* exceeding their body projection area $S=l^{2}$ by many orders of magnitude, H≫S.

Importantly, these bigger locomotive organisms must feed on live plant biomass. Indeed, at any moment of time, they occupy only a tiny portion S/H≪1 of their feeding territory and thus cannot compete for dead plant biomass with the immobile heterotrophs (bacteria and fungi) that are ubiquitously present. This explains the pattern noted by Cebrian [77] that vertebrate (i.e., bigger) detritivores are rare and their role is insignificant. The smallest heterotrophs, on the other hand, should preferentially consume dead plant biomass (i.e., they should be detritivorous) because live plants defend themselves biochemically, and it would be costly to overcome their defense. The bigger organisms have no choice but to accept these costs.

Plants represent the energetic basis for ecosystem functioning—indeed, *“the removal of primary producers leaves no system at all”* [78]. As the bigger organisms consume live plant biomass, the big herbivores as an ecological and evolutionary group present a persistent threat to ecosystem stability. Fires also consume live plant biomass at a rate that is comparable to herbivores in disturbed ecosystems (Table 1, column 6) representing some of the more voracious “plant-feeders” [79]. However, unlike living organisms, fires do not evolve. Thus, a natural ecosystem can in principle evolve to a state where it becomes resistant to fires “once and for all”, by employing efficient water retention and moisture transport mechanisms. Meanwhile any resistance the plants would evolve against herbivores, e.g., [79] Figure 2d,e, can be in principle overcome by evolution of the big herbivores themselves. This does not mean that any big herbivore the evolution has produced will destroy its own ecosystem by overfeeding. However, there are no general ecological, biological, or physical laws that would prohibit the occasional self-destructive increase in population numbers of big herbivores.

Persistent, stable ecosystems, both on land and in the ocean that apparently managed to keep population densities of big herbivores in check allocate a very low portion of total ecosystem flux to the biggest organisms (Figure 4b). The largest share of ecosystem productivity (80–90%) goes via the detrital channel of the smallest heterotrophs, bacteria, fungi, and eukaryotic unicells. These organisms keep their mass-specific metabolic rates within the optimal range (Figure 2) and do not exceed the critical linear size $l_{c}$ associated with ecological instability (Figure 4a). Together with plants, they represent the main working unit of life whose functioning is stabilized in accordance with the law of large numbers.

Invertebrates of intermediate size are still metabolically optimal (Figure 2), but their size is already supercritical (Figure 4a). These organisms are allocated a smaller share of about 10% of ecosystem energy flux (Figure 4b). Finally, the biggest organisms, vertebrates, have fallen out of the optimal metabolic range and increased their body size by two orders over the critical $l_{c}$. This double suboptimality is associated with the lowest share of ecosystem energy flux (less than 1%) allocated to these organisms in stable conditions (Figure 4b).

Recently in a discussion of the ecological role of big animals opened in the Russian Journal of Ecosystem Ecology, e.g., [75,79,80,81,82,83,84,85] Hatton and Galbraith [86] suggested that the idea of an ecological and environmental disruption associated with big animals might be in contradiction with the approximate constancy of population energy consumption observed in different-sized vertebrate species, as established by Hatton et al. [69] [their Figure 2E]. To address this criticism, we first note that the pattern shown in Figure 4b is consistent with the data of Hatton et al. [69] and Hatton and Galbraith [86]. Indeed, share β of ecosystem energy flux consumed by all organisms in a given body size interval is equal to

(3) $$\beta =\sum_{i=1}^{n}\beta_{i}=\frac{\sum_{i=1}^{n}N_{i}Q_{i}}{P},$$

where $N_{i}$ (m${}^{-2}$) is population density of the *i*-th species, *Q* (W) is whole-body metabolic rate of individuals in this species, $\beta_{i}=N_{i}Q_{i}/P$ is population energy consumption of the *i*-th species, *n* is the total number of species in the considered body size interval, and *P* (W m${}^{-2}$) is ecosystem primary productivity. If population energy consumption $\beta_{i}$ is independent of body size, as Figure 2E of Hatton et al. [69] suggests, the decline of β with increasing body size will occur at the expense of the declining number *n* of the bigger species (e.g., in most ecosystems, there are more species of rodents than of big ungulates), as also recognized by Hatton and Galbraith [86]. (Note that if $N_{i}$ is expressed in ind m${}^{-2}$, *Q* should be expressed in W ind${}^{-1}$ and body mass *m* in kg ind${}^{-1}$ such that the relationship between whole-body *Q* and mass-specific *q* metabolic rates, Q=qm, is preserved.)

Furthermore, any global compilation of $\beta_{i}=N_{i}Q_{i}/P$ necessarily includes population density data from significantly perturbed ecosystems including some overgrazed nature reserves in Africa [87]. In such ecosystems, population energy consumption by big animals can be artificially elevated by providing extra food to the protected species. Controlling for the degree of anthropogenic disturbance may change the size-related pattern for $\beta_{i}$ from the current size-invariance towards lower values at larger body sizes. Makarieva et al. [87] using the data of Damuth [71] showed that the scaling of $N_{i}$ is different in open (savanna, presumably less stable) versus closed (forests, presumably more stable) ecosystems: in closed canopy ecosystems, $N_{i}$ declines more rapidly with growing body size.

(We note in passing that in their pan-eukaryotic compilation of $N_{i}Q_{i}$ estimates Hatton et al. [69] included multicellular plants, which, unlike the remaining species of the biosphere, are known to contain large amounts of metabolically inactive structural tissues (wood). This raises the problem of defining the metabolically relevant whole-plant mass, which is smaller than total plant mass. Relocating the relatively high plant $\beta_{i}$ towards smaller body sizes in Figures 2E and 1C of, respectively, Hatton et al. [69] and Hatton and Galbraith [86], would have made the overall dependence of $N_{i}Q_{i}$ on body size across all eukaryotes more negative.)

Finally, the logic of Hatton and Galbraith [86]’s objection that big animals are not dangerous since their share of consumption declines with body size such that they may not claim all ecosystem productivity does not contradict the statement that big animals are potentially dangerous. As long as big animals are confined within the ecological corridor of relatively low β as outlined in Figure 4b, they do not pose a threat to the ecosystem functioning; the ecosystem is balanced. However, since big herbivores are physiologically selected to destroy live plants, it appears to be just a matter of time when a big species with a sufficiently destructive potential arises in the course of evolution. *Homo sapiens* appears to be a conspicuous example [88].

## 5. Anthropogenic Energy Consumption

While there is no general increase in energy rate density *q* in plants and animals to *“parallel the emergence of major evolutionary stages on the scale of life’s history”* cf. [15], the human civilization does consume a relatively large amount of energy per unit time per unit live mass. For 2015, the global energy consumption $P_{c}=17\times 10^{12}$ W (550 quadrillion btu per year) [43] divided by cumulative mass of all human bodies $M_{h}=4\times 10^{11}$ kg [46] gives $q_{c}=P_{c}/M_{h}=43$ W kg${}^{-1}$ for the modern civilization. This is more than an order of magnitude higher than a typical $q_{h}\approx 1.5$ W kg${}^{-1}$ of the human body. In countries consuming several times more energy power than the global average, the ratio $q_{c}/q_{h}$ may well reach 200.

Do those high energy rate densities, $q_{c}$∼$10^{2}$ W kg${}^{-1}$, testify for an increased complexity and evolutionary advancement our civilization could be proud of? Our species appears to be using so much energy because a certain part of the *Homo sapiens* population was historically forced out of their natural ecological niche (a warm tropical climate with a high biological productivity). With technological progress they have attempted to recreate the “paradise” conditions in a significantly more hostile and less productive environment of the higher latititudes [14]. This requires a lot of energy.

However, such elevated rates are not unprecedented in the living world. Growing bacteria and animal tissues during peak activity, e.g., during flight, consume energy at rates of up to several thousand Watts per kilogram [89]. However, living organisms do not normally maintain such rates for a prolonged period of time. This indicates that such levels of energy consumption are not sustainable in the long term.

Likewise, while global rates of synthesis and decomposition of organic matter in the biosphere balance each other with a high accuracy ensuring life’s persistence in the long term, the energy consumption of the modern civilization mostly derives from fossil fuels that are not renewable. All major mineral and energy resources that our civilization consumes at an ever increasing rate are in decline. Unlike in the rest of life, here we are apparently dealing with an unsteady process of an explosion-like exponentially accelerating energy consumption that will end in the depletion of energy resources and the collapse of the corresponding energy-consuming structure. The instantaneous unsteady rate of energy consumption can be significantly higher than our civilization’s $q_{c}$ in many processes like nuclear bomb explosions that cannot claim high orderliness (Table 1). Biospheric processes, in contrast, are characterized by a slow-down of potentially explosion-like processes. In particular, the biotic control of water vapor condensation in natural forests stabilizes the terrestrial water cycle against such weather extremes with explosion-like energy release as hurricanes and tornadoes [39,40,42].

Per unit surface area, the energy consumption of our civilization $J_{c}=P_{c}/S_{E}=0.03$ W m${}^{-2}$, $S_{E}=5\times 10^{14}$ m${}^{2}$, is about an order of magnitude lower than the global gross photosynthetic power on Earth $FK_{C}/S_{E}$∼0.6 W m${}^{-2}$. However, it exceeds the ecologically permissible energy quota for animals of human size by more than an order of magnitude (Figure 4b), illustrating the significant disruptive potential of our species with respect to destabilising the biosphere. (Direct consumption of primary productivity of the biosphere (food, cattle fodder, wood) by humans appears, in power units, to be of the same order of magnitude as consumption of fossil fuels which mostly determines $J_{c}$ [90]. Fires provide a similar disturbance removing annually about 4% of net primary productivity on land (Table 1).)

The structure of anthropogenic energy consumption reveals its low information content. About one third of all energy flow is spent on transport. Transport vehicles represent macroscopic objects whose behavior can be characterized by just a few variables like the velocity vector of its movement. Its environmental function consists of consuming petrol and releasing carbon dioxide. In contrast, the continuous cover of plants and immobile heterotrophs covering the Earth’s surface consists of microscopic memory cells that independently censor environmental conditions on a fine scale and react to environmental perturbations in a non-random way. Each such cell possesses an information-processing capacity comparable with that of a modern personal computer [7]. For example, the functioning of soil biota can change the life-time of organic compounds turning normally short-lived substances like proteins (that rapidly degrade under laboratory conditions) into long-lived ones and vice versa [91]. The meaning of *“Once there were bacteria, now there is New York”* may be precisely the opposite to what this phrase is usually invoked to imply [5].

Generally, living organisms impose control over their external environment via the surface of their bodies. With the growing size of compact animal bodies, the relative number of body cells that are in contact with the external milieu diminishes inversely proportionally to linear body size *l*:

(4) $$\eta =\frac{S}{s}\frac{v}{V}=\frac{l_{\mathrm{cell}}}{l},$$

where $S=l^{2}$ and $V=l^{3}$ are the animal body area and volume, $s=l_{\mathrm{cell}}^{2}$ and $v=l_{\mathrm{cell}}^{3}$ are the mean area and volume of cells in the animal body.

For large animals cells that interact with the external environment represent a negligible proportion of total body cells, e.g., $\eta =10^{-4}$ for l=0.5 m and $l_{\mathrm{cell}}=50\;\mu$m. Information flux in the animal body is proportional to total energy consumption of the animal. The larger the animal, the smaller the share of its information flux it can spend to participate in the biotic regulation of the external environment which allows for its very existence. Large animals use the available fluxes of energy and information almost exclusively to maintain the orderliness of their internal milieu rather than the external environment [14]. Failure to process environmental information at a sufficient rate leads to ecological misbehavior and demise. In certain aspects, this can be compared to the economic phenomenon of a decline of big monopolies that focus too much on themselves while paying insufficient attention to, and failing to predict, the market trends their smaller competitors eagerly monitor [92].

**Figure 5:**
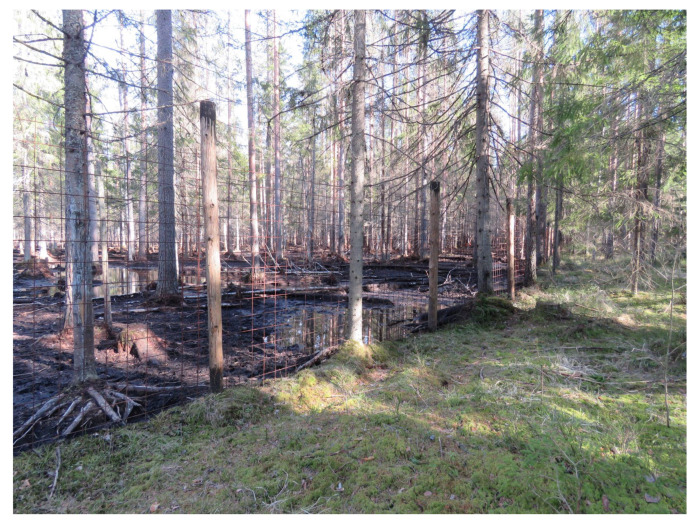
Degradation of vegetation in a forest enclosure for wild boars who are supported by extra fodder. Russia, Leningrad district, 2020. Photo by A. Nefiodov.

When large animals begin to dominate the ecosystem, the environment left without biotic control rapidly degrades. As it degrades, so does the efficiency of competitive interaction, which can no longer stabilize the genetic information of life. Thus, the spontaneous occurence, in the course of evolution, of yet bigger life’s forms may not represent some evolutionary advancement, but, on the contrary, can be manifestation of degradation (ageing) of life’s algorithm as a whole. Life is trying to counteract this decay process by limiting the energy resources allocated to big animals (Figure 4b). When these limits are overcome by whatever reason, the environment degrades locally (Figure 5) or globally depending on the spatial scale where the limits have been broken.

## 6. Methods

The dependence reported by Wright et al. [64] Table 1 and Supplementary Information, $\log_{10}\left(q_{d}/\bar{q_{d}}\right)=-1.04\log_{10}\left(\mathrm{LMA}/\bar{\mathrm{LMA}}\right)$, where $q_{d}$ and LMA are, respectively, the rate of dark respiration per unit dry leaf mass and dry leaf mass per unit leaf surface area, $\bar{q_{d}}=9.7$ nmol O${}_{2}$ (g dry mass)${}^{-1}$ s${}^{-1}$, $\bar{\mathrm{LMA}}=128$ (g dry mass) m${}^{-2}$, corresponds to $\log_{10}\left(q/\bar{q}\right)=-1.04\log_{10}\left(l/\bar{l}\right)$, where *q* is the rate of dark respiration per unit live mass and *l* is leaf thickness estimated from LMA, $\bar{q}=1.45$ W kg${}^{-1}$, $\bar{l}=4.3\times 10^{-4}$ m. (Assuming 30% dry mass content in live mass and live matter density $\rho =10^{6}$ g m${}^{-3}$, we have *l* [m] = LMA/0.3 [(g dry mass) m${}^{-2}$]/ρ; assuming 20 J per 1 ml O${}_{2}$ [58] we have *q* [W kg${}^{-1}$] = $0.15q_{d}$ [nmol O${}_{2}$ (g dry mass)${}^{-1}$ s${}^{-1}$].)

The data for starved Protozoa from Ikeda [63], Table S1-1 (thin red line in Figure 2) were corrected to 25 ${}^{\circ }$C assuming $Q_{10}=2$ as in Makarieva et al. [58] by multiplying the original metabolic rates by $2^{\left(25^{\circ }C-T\right)/10^{\circ }C}$, where *T* is the measurement temperature in degrees Celsius. Note that, in Table S1-1 of Ikeda [63], the wet mass of cells is mistakenly represented in nanograms ($10^{-6}$ mg), it should be picograms instead ($10^{-9}$ mg) to match the (correct) values for cell volumes. Data for starved cells correspond to those rows in Table S1-1 of Ikeda [63], where variables “GR” and “UNS” are both zeros.

The violet solid line in Figure 2 describes five mammalian species where oxygen consumption of the brains was measured in vivo [65] Figure 1, the violet dashed lines also include the remaining indirectly obtained estimates from Table 1 of Mink et al. [65].

In Figure 3, lizard population densities *N* (m${}^{-2}$, an inverse area per one individual) and body masses *m* were taken from Appendix S1 of Novosolov et al. [70] (148 island and 198 mainland populations). Mammalian population densities *N* and body masses *m* were taken from the Supplementary Data of Hatton et al. [69] (2663 populations for 608 species). Given the highly uneven numbers of populations studied for different mammal species, a mean population density for each mammalian species was estimated by taking a geometric mean of all population densities for the considered species. To obtain population energy consumption J=NQ (W m${}^{-2}$), each lizard population density and each mean mammalian population density were multiplied by field metabolic rate $Q_{f}$ (W) as established by Nagy [93] for reptiles, $Q_{f}/Q_{0}=0.196{\left(m/m_{0}\right)}^{0.889}$, and mammals, $Q_{f}/Q_{0}=4.82{\left(m/m_{0}\right)}^{0.734}$, respectively, where $Q_{0}=1\;\mathrm{kJ}\;{\mathrm{day}}^{-1}=0.0116\;W$, $m_{0}=1$ g.

Note the relationship between population energy consumption J=NQ and individual surface-specific energy consumption j=ρql=Q/S (Figure 4a), where $S=l^{2}$ is the projection area of the individual on the ground surface: J=jSN.

## 7. Discussion and Conclusions

We have considered several key statements of the biotic regulation of the environment that views life and environment as inseparable entities. In closed non-equilibrium systems, entropy diminishes. In open non-equilibrium systems that consume ordered energy, entropy may remain constant and the systems can be stabilized in the non-equilibrium state as long as this energy inflow is available. For example, as long as solar energy generates the fluxes of evaporation from the oceanic surface, there will be hurricanes.

However, for living systems, their order (measured as the stabilization of the genetic information against decay) is not directly related to the availability of external energy supply. If the organism does not eat, it will die. However, even if it does eat, it will die. This allows the formulation of a constraint on the information entropy [6]: if the external energy flow is low ordered compared to the information carrier, the information content can be stabilized if and only if this information carries a program of such stabilization. The measure of how disordered external energy is as compared to the ordered object in question is provided by the probability of spontaneous arisal of this object in the presence of this energy. Unlike hurricanes, living objects never arise spontaneously.

The genetic program of life must carry a subunit about how to recognize and delete the copies with erroneous information. The process of recognition of erroneous copies represents a measurement. This measurement is performed by classical objects (individuals) that all age and die. The deletion of erroneous copies must involve their deprivation of essential resources like energy. Thus, the efficiency of the stabilization program depends on the environmental properties (Figure 1). Under conditions of unlimited resource abundance, the deletion of erroneous copies (and defective individuals) from the population would be impossible [28]. Individuals incapable of environmental regulation will propagate at the expense of consuming the available resources until they force out all “regulators”, after which the biotic regulation discontinues and the environment degrades.

A theoretical question is whether it is possible to conceive a program that will sustain itself eternally (provided the solar energy flux and other basic planetary characteristics remain constant). This is not obvious. The appearance of big animals, and generally the body size growth in the course of evolution, shows that, when the cells of the multicellular body act in concert (mutually correlated), they can at least sometimes outcompete the small organisms that represent independent individuals. This increase in individual competitive capacity comes hand in hand with the ability of large animals to destroy live plant biomass (Figure 5).

Apparently, both in the ocean and on land, the large-scale evolutionary increase in body size—from unicellular organisms to multicellular invertebrates to yet bigger vertebrates—was paralleled with a decline of the share of ecosystem energy consumption allocated to them—from, respectively, ∼100% to ∼10% to ∼1% (Figure 4b). It is most pronounced in the ocean [75], where there are no large primary consumers: the biggest animals reside at the top of the food pyramid with extremely low population energy consumption.

The energetic deprivation of the biggest animals, and the destabilization their high population numbers can invoke, suggest that the spontaneous increase in body size during life’s evolution may represent a drawback of the life’s algorithm as a whole, a sign of its ageing and losing order. Given that the key function of life is to maintain an environment where it can stabilize itself, big animals with their low surface-to-volume ratio are unable to perform efficient environmental regulation but are potentially capable of destruction.

Brains, and the human brain in particular, are tissues that display a close to optimum volume-specific energy rate density (Figure 2). Apparently, having discovered external energy resources, the humanity has found itself in a temporary state of energy abundance and has been so far disturbing and destroying the biosphere. However, it is in principle possible that with use of their metabolically optimal brains humans will realize the principles of life stability and implement them into their culture. The danger of resource abundance for population organization has been intuitively recognized in some cultures, whereby the belongings of the deceased are destroyed preventing the accumulation of wealth [94]. However, a scientific recognition of the danger of abundance is required. If this is done, then the degradation of life could be stopped via human knowledge.

The biotic regulation concept gives meaning to the information content of the genome: the information makes sense if it fits the general program of keeping the environment stable. This information can be copied infinitely without loss as it is written on quantum carriers (DNA molecules) that do not age. The combination of quantum and classical properties is a distinct feature of life as compared to processes in the inanimate nature. In modern computers, programs are written on macroscopic non-quantum memory cells. Errors that arise in these programs do not obey the quantum law of radioactive decay. This copying does not necessarily produce identical copies. Technical problems or improper handling of physical data carriers can lead to disappearance of all copies. However, program users are able to recognize and delete erroneous, virus-affected programs and by doing so they act as the classical individuals in the natural biota.

The modern civilization is in this context fully classical. It does not contain any quantum program of the stationary norm and how to prevent decay. Life, on the other hand, does not contain any information about the stationary state of the civilization. In the inanimate nature, there cannot be sustainable development; there is only the decay of an ordered state that determines the direction of time. The irreversibility of time is manifested in the impossibility of creating perpetuum mobiles of the second kind—one cannot get work from heat at the same temperature. In the living nature there is only one example of sustainable development—it is the programmed development of an embryo from the egg or a plant from the seed. This development occurs strictly in line with the genetic program of the species that determines the decay channels for energy transformations; and which processes must occur at each stage. The normal process of development, as well as deviations from the norm, are well defined. This sustainable development according to the genetic program formed during the billions of years of biological evolution culminates in the stationary life stage of the adult individual. This life continues for a known period of time and ends with the decay and death of the individual. For most species, the biosphere provides sustainable conditions for the development of all generations of organisms for several million years.

In contrast to biological species, human civilization does not have a program of sustainable development. Such a program can be created only on the basis of fundamental research. In a broad sense, sustainable development of the civilization can be understood as a steady-state process of cultural change: the civilization continues to evolve culturally (in particular, scientifically and technologically), but the environmental conditions persist that enable all generations to thrive [95,96,97]. Currently, attempts at climate stabilization are primarily based on technology and aimed at mitigating carbon dioxide emissions. Meanwhile, protection and conservation of natural ecosystems are viewed as a related but separate goal. Reflecting this duality, there are different international organizations dealing with the presumably different sets of problems, e.g., the Intergovernmental Panel on Climate Change and the Intergovernmental Science-Policy Platform on Biodiversity and Ecosystem Services [98]. The biotic regulation concept indicates that environmental sustainability cannot be achieved without understanding and quantifying the genetically encoded regulatory functions of the natural ecosystems. A wide international effort is needed to study the stabilizing climate impact of the still remaining least disturbed natural ecosystems [99,100,101]. Long-term climate stability, and thus sustainability on a planetary scale, is contingent upon the existence of vast areas where natural ecosystems are allowed to operate maintaining the environmental conditions in a suitable for life state.

## Figures and Tables

**Table 1:** “Explosion” and “slow-down” energy consumption patterns in the biota and civilization.

	Long-Range Fusion Reactions				Electromagnetic Long-Range						Gravitational Long-Range			Short-Range Decay	
	in Production of Photons				Interaction						Interaction			of Uranium	
	Civilization	Sun			Civilization			Biota			Abiotic	Biota		Civilization	
	Explosion ^1^	Slow-Down ^2^	Slow-Down ^3^		Explosion ^4^	Slow-Down ^5^		Explosion ^6^	Slow-Down ^7^		Explosion ^8^	Slow-Down ^9^		Explosion ^10^	Slow-Down ^11^
	Tsar	on the	on the		TNT	global		disturbed	intact		storms,	biotic		Little	nuclear
	Bomba	Sun	Earth		(wars,	energy		ecosystems	forests		floods	pump		Boy	reactors
			orbit		mining)	usage									
Storage															
per unit	$10^{13}$	$10^{14}$	0		$10^{7}$	$10^{7}$		$4\times 10^{6}$	$4\times 10^{6}$		storms	$2\times 10^{3}$		$10^{12}$	$10^{12}$
mass,			(massless								$10^{4}$				
J kg${}^{-1}$			photons)								floods				
											50				
Total	$10^{17}$	$10^{44}$	0		$10^{13}$	$10^{20}$		$4\times 10^{21}$	$10^{21}$		storms	$10^{20}$		$10^{14}$	$10^{20}$
storage, J			(massless								$10^{17}$				
			photons)								floods				
											$10^{16}$				
Local	$10^{18}$				$10^{18}$	cars,		fires	fires		storms	20		$10^{15}$	$10^{8}$
power						heating,		$10^{4}$	$10^{4}$		$10^{6}$				
per unit						industry		herbivores	herbivores		floods				
surface						$10^{3}$		$10^{3}$	$10^{3}$		0.6				
area, W m${}^{-2}$															
Mean		$6\times 10^{7}$	340		$10^{4}$	cars,		fires	fires		storms	2			$10^{-3}$
power						heating,		0.02	$10^{-4}$		0.5				
per unit						industry		herbivores	herbivores		floods				
surface						$10^{5}$		0.05	0.005		$10^{-4}$				
area, W m${}^{-2}$															
Total	$2\times 10^{25}$	$4\times 10^{26}$	$1.7\times 10^{17}$		$10^{19}$	$1.7\times 10^{13}$		fires	fires		storms	$2\times 10^{13}$		$10^{20}$	$10^{12}$
(global )						electricity		$3\times 10^{12}$	$10^{9}$		$3\times 10^{14}$				
power,						$10^{12}$		herbivores	herbivores		floods				
W								$7\times 10^{12}$	$7\times 10^{10}$		$10^{10}$				
Decay	$10^{-8}$ s	$10^{10}$	$10^{10}$		$10^{-6}$ s	3		1	$10^{3}$		10	10		$10^{-6}$ s	40
time		years	years			months		day	years		days	days			years

${}^{1}$ Thermonuclear explosion of the Soviet hydrogen bomb (Tsar Bomba) AN602. Mass is 26.5 t, measured blast yield is about 58.6 Mt of trinitrotoluene (TNT) equivalent ($2.4\times 10^{17}$ J). The corresponding mass defect of 2.65 kg is spent on production of massless photons. The density is assumed to be equal to the Sun’s average density, which is close to the density of liqud water. Decay time is estimated as the time of light propagation throughout the bomb volume with the characteristic length of 5 m: ∼5 m/[$3\times 10^{8}$ m s${}^{-1}$]∼$10^{-8}$ s. Total power is given by $2.4\times 10^{17}$ J/[$10^{-8}$ s]∼$2\times 10^{25}$ W. Initial radius of explosion is taken as 4.6 km. Specific power of energy flux through a unit of surface area at the beginning of explosion is about $2\times 10^{25}$ W/[4.6 km]${}^{2}$∼$10^{18}$ W m${}^{-2}$. ${}^{2}$ The data are taken from [45]. Note that the total power of thermonuclear explosion of the Tsar Bomba is just about 20 times lower than the power output from the Sun’s surface. The only known way to slowdown the thermonuclear reaction is to arrange the thermonuclear fuel within the Sun’s volume. ${}^{3}$ The data are taken from [45]. ${}^{4}$ Average energy store per kilogram is twice as much as that in conventional fuel (1 kg of TNT∼$4.2\times 10^{6}$ J). The strategic stockpile of explosives in various forms is assumed to correspond to three months of use for explosions. The global average (and local) power of explosions is determined by using the time of explosion and average mass (∼1 kt) of the TNT exploded. The global power of TNT explosions is $10^{13}$ J/[$10^{-6}$ s] $=10^{19}$ W. ${}^{5}$ Energy store per kilogram of organic substance has the same order of magnitude as that in the fourth column. The global supply of fossil fuels is determined by the power consumption under the assumption that the strategic reserves of fossil fuels are equal to the 3-month consumption by civilization. Local and average power per unit of Earth’s surface area is assumed to be equal to the power of cars; energy consumption of heating and industrial production are characterized by the same order of magnitude. The global power consumption of fossil fuels is known to be equal to $1.7\times 10^{13}$ W. ${}^{6}$ In columns 6 and 7, energy consumption of plant biomass is considered with energy content $K=4\times 10^{6}$ J kg${}^{-1}$ and global store of the order of $10^{15}$ kg [46] assuming most land ecosystems are disturbed; fires consume 2 GtC yr${}^{-1}$ [47], which constitutes 4% of primary productivity on land 56 GtC yr${}^{-1}$ [48] ($7\times 10^{13}$ W), which amounts to $3\times 10^{12}$ W globally or 0.02 W m${}^{-2}$ for land on average. Wood consumption due to logging consumes globally about 0.3 GtC yr${}^{-1}$ [49]; however, in many regions of the world, logging facilitates forest fires [47,50,51,52]. Instantaneous power for forest fire is estimated as the power of blackbody radiation at 800 K as $10^{4}$ W m${}^{-2}$. Decay time for fires, $10^{5}$ s = 1 day, is estimated as local biomass store in forests (∼200 kg m${}^{-2}$) multiplied by $K=4\times 10^{6}$ J kg${}^{-1}$ and divided by instantaneous local fire power; instantaneous local power of energy consumption by big herbivores, $10^{2}$–$10^{3}$ W m${}^{-2}$, is taken from Figure 4a; their total energy consumption in unstable ecosystems may reach 100% primary productivity with a global average of 10% [28]. ${}^{7}$ In intact forests, fires are suppressed by vegetation cover controlling the water regime and occur less frequently than once in a thousand years compared to disturbed ecosystems when burning may occur every 10 years. This is reflected in the mean value of burnt areas, which for non-anthropogenic fires is two orders of magnitude smaller than for anthropogenic fires [50,51]. Big herbivores in intact forests are allocated less than 1% of primary productivity (Figure 4b). Global estimates are made assuming 10% of primary production on land from intact ecosystems. ${}^{8}$ Gravitational long-range interactions (via buoyancy and condensation-induced atmospheric dynamics [53]) determine atmospheric dynamics. Explosion-like energy release in storms (hurricanes and tornadoes) is not controlled by the biota. The energy storage per unit mass is estimated from volume-specific energy storage $\rho_{a}V^{2}=10^{4}$ J m${}^{-3}$, where *V*∼$10^{2}$ m s${}^{-1}$ is wind velocity in the eyewall of the storm and $\rho_{a}=1$ kg m${}^{-3}$ is density of atmospheric air. Instantaneous power per unit surface area for storms is estimated as $\rho_{a}V^{3}=10^{6}$ W m${}^{-2}$. Energy storage in floods is gh=50 J kg${}^{-1}$, where *h*∼5 m is height of the surge, g=9.8 m s${}^{-2}$ is the gravitational acceleration. Assuming that during the flood the water rises by *h*∼5 m in t=5 days, the local flood power is ρgh(h/t)=0.6 W m${}^{-2}$, where $\rho =10^{3}$ kg m${}^{-3}$ is density of liquid water. Mean global power for floods is estimated assuming that every flood lasts about 2t=10 days and in one year the total globally affected area is about $S_{fl}=10^{6}$ km${}^{2}$ [54], $\rho gh\left(h/t\right)\left(2t/\left[1\;\mathrm{year}\right]\right)S_{fl}=10^{10}$ W. Total energy storage in floods $\rho gh^{2}\left(2t/\left[1\;\mathrm{year}\right]\right)S_{fl}=10^{16}$ J. Total energy storage in storms is estimated assuming a storm lasting on average 10 days and there being globally 40 storms each year [55] with total energy $\rho_{a}V^{2}HL^{2}=10^{17}$ J, where H=10 km is height of the atmosphere and L=100 km is storm’s radius where V≳30 m s${}^{-1}$. Total storm power is $\rho_{a}V^{3}L^{2}=3\times 10^{14}$ W, global surface-specific power density divides this number by the Earth’s surface area $S_{E}$. ${}^{9}$ The biotic pump mechanism regulates precipitation and winds. The dry air can ascend and descend in the atmosphere of Earth with zero net power expenditure; in contrast, water vapor condenses as it ascends, disappears from the gas phase and returns to the ground surface in the liquid or solid form. This water vapor disappearance from the gas phase creates a vertical pressure gradient (a deviation from the hydrostatic equilibrium) that acts as a vertical force pushing the air upward. Work of this force per unit time represents the power of the biotic pump air circulation [53]. The nearly instantaneous restoration of the hydrostatic equilibrium of the air occurs at the expense of the horizontal air inflow, mostly from the adjacent oceans. In a steady state, this incoming flow of moist air increases the precipitation rate over the forests by the magnitude of the river runoff. How to harmonize the fluxes of evaporation and precipitation such that their difference is equal to the amount of moisture needed to compensate the river runoff–at the same time minimizing the occurrences of hurricanes, tornadoes, floods and droughts–represents one of the most complex processes of the biotic regulation of the environment. In intact forests, fluctuations of precipitaton are suppressed by the biotic pump mechanism [41,42]. The energy store in the atmosphere is given by $p_{v}/\rho_{a}=2\times 10^{3}$ J kg${}^{-1}$, where $p_{v}$∼$2\times 10^{3}$ Pa is partial pressure of atmospheric water vapor. Total store is given by the product of $p_{v}h_{\gamma }S_{fo}=10^{20}$ J, where $h_{\gamma }=5$ km is the scale height for the vertical distribution of relative partial pressure of water vapor in the gravitational field [53] and $S_{fo}=10^{13}$ m${}^{2}$ is the total global area of intact forest landscapes [56]. Decay time is the turnover time of water in the atmosphere. Mean power of wind associated with condensation rate controlled by forests is ΠRT=2 W m${}^{-2}$, where Π=0.5 m yr${}^{-1}=10^{-3}$ mol H${}_{2}$O s${}^{-1}$ m${}^{-2}$ is mean precipitation rate on land, R=8.3 J mol${}^{-1}$ K${}^{-1}$ is the universal gas constant and *T*∼300 K is absolute temperature. The estimate of local power assumes that precipitation events on average take about one tenth of the time. ${}^{10}$ Characteristics are estimated of the atomic bomb (Little Boy) dropped on Hiroshima on 6 August 1945. The bomb with mass of 4.4 t contained 64 kg of enriched uranium. Blast yield is 15 kt of TNT ($63\times 10^{12}$ J). Initial radius of explosion is taken as 100 m. ${}^{11}$ The fission of one atom of uranium-235 releases on average 200 MeV ($3\times 10^{-11}$ J) with taking into account the decay of its fragments or $83\times 10^{12}$ J per kilogram of ${}^{235}$U. For low-enriched uranium (3 to 5% concentration of uranium-235), which is usually used as fuel for thermal-neutron reactors at nuclear power plants, it corresponds to the energy storage of about $3\times 10^{12}$ J per kilogram of uranium. Although uranium is quite widespread in nature, only a relatively small portion of it is concentrated in deposits that are economically profitable for industrial development and use in nuclear energetics. Identified resources in situ recoverable at a cost not exceeding 260 USD per kilogram of uranium are estimated to be ∼$10^{7}$ t [57]. Since natural uranium contains about 0.7% of fissile isotope ${}^{235}$U, total storage of energy is given by $0.007\times 10^{10}$ kg × $3\times 10^{12}$ J/kg∼$2\times 10^{20}$ J. Presently, there are about 450 nuclear power reactors in operation worldwide with generating electric-power capacity of $4\times 10^{11}$ W. The average efficiency of conversion of heat to electric power is about 30%. Total (global) power of nuclear reactors is estimated as $10^{12}$ W, while the mean power per unit surface area is $10^{12}$ W/[$5\times 10^{8}$ km${}^{2}$]∼$2\times 10^{-3}$ W m${}^{-2}$, where the Earth’s surface area $S_{E}$ is taken into account. A typical nuclear power station generates electricity at a rate of ∼$10^{9}$ W. Taking into account that a nuclear reactor core has an effective radius of 3 m, one can estimate the local surface-specific power of nuclear reactor as $10^{9}$ W/[3 m]${}^{2}$∼$10^{8}$ W m${}^{-2}$. Nuclear stations are designed for at least a 40-year operating life.

## Abbreviations

The following abbreviations are used in this manuscript:

btu	British thermal unit, 1 btu = 1055 J
DNA	Deoxyribonucleic acid
GtC	Gigaton of carbon, $1\;\mathrm{Gt}=10^{9}\;t=10^{12}\;\mathrm{kg}$
ind	Individuals
LMA	Dry leaf mass per unit leaf surface area
MeV	Megaelectronvolt, $1\;\mathrm{MeV}=10^{6}\;\mathrm{eV}=1.6\times 10^{-13}\;J$
TNT	Trinitrotoluene

## References

[1] Brillouin L. (2013). Science and Information Theory.

[2] Packard N.H., Kelso J.A.S., Mandell A.J., Shlesinger M.F. (1988). Adaptation Toward the Edge of Chaos. Dynamic Patterns in Complex Systems.

[3] Langton C.G. (1990). Computation at the edge of chaos: Phase transitions and emergent computation. Physica D.

[4] Yockey H.P. (2005). Information Theory, Evolution, and The Origin of Life.

[5] Morris S.C., Lineweaver C.H., Davies P.C.W., Ruse M. (2013). Life: The final frontier for complexity?. Complexity and the Arrow of Time.

[6] O’Connor M.I., Pennell M.W., Altermatt F., Matthews B., Melián C.J., Gonzalez A. (2019). Principles of Ecology Revisited: Integrating Information and Ecological Theories for a More Unified Science. Front. Ecol. Evol..

[7] Gorshkov V.G. (1996). Stores and flows of information in biota and civilization. Dokl. Biol. Sci..

[8] Landauer R. (1961). Irreversibility and heat generation in the computing process. IBM J. Res. Dev..

[9] Landauer R. (1996). Minimal energy requirements in communication. Science.

[10] Sagawa T., Ueda M. (2009). Minimal Energy Cost for Thermodynamic Information Processing: Measurement and Information Erasure. Phys. Rev. Lett..

[11] Sagawa T., Ueda M. (2010). Sagawa and Ueda Reply. Phys. Rev. Lett..

[12] Dillenschneider R., Lutz E. (2010). Comment on “Minimal Energy Cost for Thermodynamic Information Processing: Measurement and Information Erasure”. Phys. Rev. Lett..

[13] Kempes C.P., Wolpert D., Cohen Z., Pérez-Mercader J. (2017). The thermodynamic efficiency of computations made in cells across the range of life. Philos. Trans. R. Soc. A.

[14] Makarieva A., Gorshkov V., Wilderer P.A., Wilderer P.A., Grambow M. (2016). What Can We Learn from Natural Ecosystems to Avoid a Civilization Breakdown. Global Stability through Decentralization?.

[15] Chaisson E.J. (2015). Energy flows in low-entropy complex systems. Entropy.

[16] Koza J.R. (1992). Genetic Programming: On the Programming of Computers by Means of Natural Selection.

[17] Mitchell M., Hraber P.T., Crutchfield J.P. (1993). Revisiting the Edge of Chaos: Evolving Cellular Automata to Perform Computations. Complex Syst..

[18] Chaisson E.J. (2001). Cosmic Evolution: The Rise of Complexity in Nature.

[19] Chaisson E.J. (2011). Energy rate density as a complexity metric and evolutionary driver. Complexity.

[20] Chaisson E.J. (2014). The Natural Science Underlying Big History. Sci. World J..

[21] Gorshkov V.G., Makarieva A.M. (2001). On the possibility of physical self-organization of biological and ecological systems. Dokl. Biol. Sci..

[22] Gorshkov V.V., Gorshkov V.G., Danilov-Danil’yan V.I., Losev K.S., Makarieva A.M. (2002). Information in the animate and inanimate worlds. Rus. J. Ecol..

[23] Gorshkov V.G. (1995). Physical and Biological Bases of Life Stability. Man, Biota, Environment.

[24] Bardi U. (2020). Gaia and biotic regulation: Understanding the homeostasis of the ecosphere. Rus. J. Ecosyst. Ecol..

[25] Gorshkov V.G., Gorshkov V.V., Makarieva A.M. (2000). Biotic Regulation of the Environment: Key Issue of Global Change.

[26] Gorshkov V.G., Makarieva A.M. (2020). Quantum and Classical Aspects of Life Organization. https://www.bioticregulation.ru/ab.php?id=lifepr.

[27] Gorshkov V.G., Makarieva A.M. (2020). Time in Life, Technology and Physics. https://bioticregulation.ru/ab.php?id=time.

[28] Gorshkov V.G., Makarieva A.M. (2020). Key ecological parameters of immotile versus locomotive life. Rus. J. Ecosyst. Ecol..

[29] Lynch M. (2016). Mutation and Human Exceptionalism: Our Future Genetic Load. Genetics.

[30] Lovelock J.E. (1972). Gaia as seen through the atmosphere. Atmos. Environ. (1967).

[31] Haldane J.B.S., Huxley J., Hardy A., Ford E. (1954). The stasis of evolution. Evolution as a Process.

[32] Gould S.J. (2007). Punctuated Equilibrium.

[33] Bomfleur B., McLoughlin S., Vajda V. (2014). Fossilized Nuclei and Chromosomes Reveal 180 Million Years of Genomic Stasis in Royal Ferns. Science.

[34] Drake J.W. (1999). The distribution of rates of spontaneous mutation over viruses, prokaryotes, and eukaryotes. Ann. N. Y. Acad. Sci..

[35] Leopold A.C. (1975). Aging, Senescence, and Turnover in Plants. BioScience.

[36] Makarieva A.M., Gorshkov V.G. (2004). On the dependence of speciation rates on species abundance and characteristic population size. J. Biosci..

[37] Chanda P., Costa E., Hu J., Sukumar S., Van Hemert J., Walia R. (2020). Information Theory in Computational Biology: Where We Stand Today. Entropy.

[38] Chopra A., Lineweaver C.H. (2016). The Case for a Gaian Bottleneck: The Biology of Habitability. Astrobiology.

[39] Makarieva A.M., Gorshkov V.G., Nefiodov A.V., Chikunov A.V., Sheil D., Nobre A.D., Li B.L. (2017). Fuel for cyclones: The water vapor budget of a hurricane as dependent on its movement. Atmos. Res..

[40] Makarieva A.M., Gorshkov V.G. (2007). Biotic pump of atmospheric moisture as driver of the hydrological cycle on land. Hydrol. Earth Syst. Sci..

[41] Millán H., Rodríguez J., Ghanbarian-Alavijeh B., Biondi R., Llerena G. (2011). Temporal complexity of daily precipitation records from different atmospheric environments: Chaotic and Lévy stable parameters. J. Atmos. Res..

[42] Makarieva A.M., Gorshkov V.G., Li B.L. (2013). Revisiting forest impact on atmospheric water vapor transport and precipitation. Theor. Appl. Climatol..

[43] Newell R.G., Raimi D., Aldana G. (2019). Global Energy Outlook 2019: The Next Generation of Energy.

[44] Bologna M., Aquino G. (2020). Deforestation and world population sustainability: A quantitative analysis. Sci. Rep..

[45] Allen C.W. (1973). Astrophysical Quantities.

[46] Bar-On Y.M., Phillips R., Milo R. (2018). The biomass distribution on Earth. Proc. Natl. Acad. Sci. USA.

[47] van Marle M.J.E., Kloster S., Magi B.I., Marlon J.R., Daniau A.L., Field R.D., Arneth A., Forrest M., Hantson S., Kehrwald N.M. (2017). Historic global biomass burning emissions for CMIP6 (BB4CMIP) based on merging satellite observations with proxies and fire models (1750–2015). Geosci. Model Dev..

[48] Field C.B., Behrenfeld M.J., Randerson J.T., Falkowski P. (1998). Primary Production of the Biosphere: Integrating Terrestrial and Oceanic Components. Science.

[49] Zhang X., Chen J., Dias A.C., Yang H. (2020). Improving Carbon Stock Estimates for In-Use Harvested Wood Products by Linking Production and Consumption–A Global Case Study. Environ. Sci. Technol..

[50] Aleynikov A.A., Tyurin A.V., Simakin L.V., Efimenko A.S., Laznikov A.A. (2015). Fire history of dark needle coniferous forests in Pechora-Ilych nature reserve since second half of XIX century to present time. Sib. Lesn. Zhurnal (Sib. J. For. Sci.).

[51] Aleynikov A.A., Lisicyna O.V., Vladimirova N.A., Krylov A.M., Simakin L.V. (2017). The Impact of Availability Territory and Terrain Characteristics on Location of Burnt Areas in Dark Coniferous Forests Pechora-Ilych Nature Reserve. For. Eng. J..

[52] Aleinikov A.A. (2019). The fire history in pine forests of the plain area in the Pechora-Ilych Nature Biosphere Reserve (Russia) before 1942: Possible anthropogenic causes and long-term effects. Nat. Conserv. Res..

[53] Makarieva A.M., Gorshkov V.G., Nobre A.D., Nefiodov A.V., Sheil D., Nobre P., Li B.L. (2019). Comments on “Is condensation-induced atmospheric dynamics a new theory of the origin of the winds?”. J. Atmos. Sci..

[54] Douben K.J. (2006). Characteristics of river floods and flooding: A global overview, 1985–2003. Irrig. Drain..

[55] Wehner M., Prabhat, Reed K.A., Stone D., Collins W.D., Bacmeister J. (2015). Resolution Dependence of Future Tropical Cyclone Projections of CAM5.1 in the U.S. CLIVAR Hurricane Working Group Idealized Configurations. J. Clim..

[56] Potapov P., Yaroshenko A., Turubanova S., Dubinin M., Laestadius L., Thies C., Aksenov D., Egorov A., Yesipova Y., Glushkov I. (2008). Mapping the world’s intact forest landscapes by remote sensing. Ecol. Soc..

[57] OECD-NEA/IAEA (2019). Uranium 2018: Resources, Production and Demand (“Red Book”). The Nuclear Fuel Report 2015, 2017 & 2019.

[58] Makarieva A.M., Gorshkov V.G., Li B.L., Chown S.L., Reich P.B., Gavrilov V.M. (2008). Mean mass-specific metabolic rates are strikingly similar across life’s major domains: Evidence for life’s metabolic optimum. Proc. Natl. Acad. Sci. USA.

[59] Makarieva A.M., Gorshkov V.G., Li B.L. (2005). Revising the distributive networks models of West, Brown and Enquist (1997) and Banavar, Maritan and Rinaldo (1999): Metabolic inequity of living tissues provides clues for the observed allometric scaling rules. J. Theor. Biol..

[60] Powers W.J., Grubb R.L., Darriet D., Raichle M.E. (1985). Cerebral Blood Flow and Cerebral Metabolic Rate of Oxygen Requirements for Cerebral Function and Viability in Humans. J. Cereb. Blood Flow Metabol..

[61] Xu F., Ge Y., Lu H. (2009). Noninvasive quantification of whole-brain cerebral metabolic rate of oxygen (CMRO_2_) by MRI. Magn. Resonan. Med..

[62] Gorshkov V.G. (1981). The distribution of energy flow among the organisms of different dimensions. J. Gen. Biol..

[63] Ikeda T. (2017). An analysis of metabolic characteristics of planktonic heterotrophic protozoans. J. Plankton Res..

[64] Wright I.J., Reich P.B., Westoby M., Ackerly D.D., Baruch Z., Bongers F., Cavender-Bares J., Chapin T., Cornelissen J.H.C., Diemer M. (2004). The worldwide leaf economics spectrum. Nature.

[65] Mink J.W., Blumenschine R.J., Adams D.B. (1981). Ratio of central nervous system to body metabolism in vertebrates: Its constancy and functional basis. Am. J. Physiol..

[66] DeLong J.P., Okie J.G., Moses M.E., Sibly R.M., Brown J.H. (2010). Shifts in metabolic scaling, production, and efficiency across major evolutionary transitions of life. Proc. Natl. Acad. Sci. USA.

[67] Johnson M.D., Völker J., Moeller H.V., Laws E., Breslauer K.J., Falkowski P.G. (2009). Universal constant for heat production in protists. Proc. Natl. Acad. Sci. USA.

[68] Kiørboe T., Hirst A.G. (2014). Shifts in Mass Scaling of Respiration, Feeding, and Growth Rates across Life-Form Transitions in Marine Pelagic Organisms. Am. Nat..

[69] Hatton I.A., Dobson A.P., Storch D., Galbraith E.D., Loreau M. (2019). Linking scaling laws across eukaryotes. Proc. Natl. Acad. Sci. USA.

[70] Novosolov M., Rodda G.H., Feldman A., Kadison A.E., Dor R., Meiri S. (2016). Power in numbers. Drivers of high population density in insular lizards. Glob. Ecol. Biogeogr..

[71] Damuth J. (1993). Cope’s rule, the island rule and the scaling of mammalian population density. Nature.

[72] Myhrvold N.P. (2016). Dinosaur Metabolism and the Allometry of Maximum Growth Rate. PLoS ONE.

[73] Griebeler E.M., Werner J. (2018). Formal comment on: Myhrvold (2016) Dinosaur metabolism and the allometry of maximum growth rate. PLoS ONE.

[74] Padian K., de Ricqlès A. (2020). Inferring the physiological regimes of extinct vertebrates: Methods, limits and framework. Philos. Trans. R. Soc. B.

[75] Nefiodov A.V. (2020). Universal patterns of matter and energy fluxes in land and ocean ecosystems. Rus. J. Ecosyst. Ecol..

[76] Perissi I., Bardi U. (2020). The Empty Sea. The Future of the Blue Economy.

[77] Cebrian J. (2004). Role of first-order consumers in ecosystem carbon flow. Ecol. Lett..

[78] Hunter M.D., Price P.W. (1992). Playing Chutes and Ladders: Heterogeneity and the Relative Roles of Bottom-Up and Top-Down Forces in Natural Communities. Ecology.

[79] Sheil D. (2020). Dangerous Giants?—Large herbivores, forest feedbacks and climate tipping points. Rus. J. Ecosyst. Ecol..

[80] Geraskina A.P., Smirnova O.V., Korotkov V.N., Kudrevatykh I.Y. (2020). Productivity and content of macro-and microelements in the phytomass of ground vegetation of typical and unique taiga forests of the Northern Urals (example of spruce-fir forests of the Pechora-Ilych nature reserve). Rus. J. Ecosyst. Ecol..

[81] Chown S.L. (2020). Reflections on Victor Gorshkov’s final work—“Key ecological parameters of immotile versus locomotive life”. Rus. J. Ecosyst. Ecol..

[82] Belotelov N.V. (2020). Impact of the works of V. G. Gorshkov on the development of mathematical models of ecosystems. Rus. J. Ecosyst. Ecol..

[83] Martyushev L.M. (2020). Development or sustainability. What is most important to the Universe? (Comment on the article “Key ecological parameters of immotile versus locomotive life” by V. G. Gorshkov and A. M. Makarieva). Rus. J. Ecosyst. Ecol..

[84] Li B.L. (2020). Mass-specific versus whole-body metabolic rate in biological scaling analyses (Commentary on “Key ecological parameters of immotile versus locomotive life” by V. G. Gorshkov and A. M. Makarieva). Rus. J. Ecosyst. Ecol..

[85] Gavrilov V.M. (2020). Territoriality and flock formation as possible mechanisms for maintaining species stability: Commentary on V. G. Gorshkov, A. M. Makarieva (2020) “Key ecological parameters of immobile and locomotive life”. Rus. J. Ecosyst. Ecol..

[86] Hatton I., Galbraith E. (2020). Commentary on “Key ecological parameters of immotile versus locomotive life” by V. G. Gorshkov and A. M. Makarieva. Rus. J. Ecosyst. Ecol..

[87] Makarieva A.M., Gorshkov V.G., Li B.L. (2004). Body size, energy consumption and allometric scaling: A new dimension in the diversity—Stability debate. Ecol. Complex.

[88] Hern W.M. (1999). How Many Times Has the Human Population Doubled? Comparisons with Cancer. Popul. Environ..

[89] Makarieva A.M., Gorshkov V.G., Li B.L. (2005). Energetics of the smallest: Do bacteria breathe at the same rate as whales?. Proc. R. Soc. B.

[90] Gorshkov V.G., Dol’nik V.R. (1980). Energetics of the biosphere. Sov. Phys. Usp..

[91] Schmidt M.W.I., Torn M.S., Abiven S., Dittmar T., Guggenberger G., Janssens I.A., Kleber M., Kögel-Knabner I., Lehmann J., Manning D.A.C. (2011). Persistence of soil organic matter as an ecosystem property. Nature.

[92] Nowlan E. (2018). X-Inefficiency in Monopolies.

[93] Nagy K.A. (2005). Field metabolic rate and body size. J. Exp. Biol..

[94] Venbrux E. (2007). Destroyal of the personal belongings of the deceased. J. de la Société des Océanistes.

[95] Brundtland G.H. (1985). World Commission on environment and development. Environ. Policy Law.

[96] Redclift M. (2005). Sustainable Development (1987–2005): An Oxymoron Comes of Age. Sustain. Dev..

[97] Purvis B., Mao Y., Robinson D. (2019). Three pillars of sustainability: In search of conceptual origins. Sustain. Sci..

[98] Brooks T.M., Lamoreux J.F., Soberón J. (2014). IPBES ≠ IPCC. Trends Ecol. Evol..

[99] Morozov V.E., Aleinikov A.A., Smirnova O.V., Anapolsky A.B., Vasilov R.G., Gavrilov V.M., Korotkov V.N., Makarieva A.M., Nefiodov A.V., Chikunov A.V. (2019). New paradigm of state policy in the field of ecology and environment and climate protection. Energy Econ. Technol. Ecol..

[100] Funk J.M., Aguilar-Amuchastegui N., Baldwin-Cantello W., Busch J., Chuvasov E., Evans T., Griffin B., Harris N., Ferreira M.N., Petersen K. (2019). Securing the climate benefits of stable forests. Clim. Policy.

[101] Watson J.E.M., Evans T., Venter O., Williams B., Tulloch A., Stewart C., Thompson I., Ray J.C., Murray K., Salazar A. (2018). The exceptional value of intact forest ecosystems. Nat. Ecol. Evol..

